# Impedance Measures and a Mounting Technique for Drosophila: Larval Movements, Heart Rate, Imaging, and Electrophysiology

**DOI:** 10.3390/mps3010012

**Published:** 2020-01-24

**Authors:** Noah de Castro, Robin Lewis Cooper

**Affiliations:** 1 Lafayette Senior High School, Lexington, KY 40503, USA; ndecastro333@gmail.com; 2 Department of Biology, University of Kentucky, Lexington, KY 40506-0225, USA

**Keywords:** impedance, gluing, Drosophila, heartrate, locomotion, optogenetics

## Abstract

Monitoring movements of larval *Drosophila* with electrical detection allows one to record the behaviors without the use of lights and cameras. This is a suitable technique when studying the use of light-sensitive proteins in optogenetic studies. Electrical measures are feasible to use in determining when a larva starts to move or continues to move after a light induced activation of channelrhodopsin. We have developed a technique using an electrical measure of the media as an index of larval movement. As a proof of concept, recordings with an infrared camera of the larval movement were simultaneous made with electrical measures. The two techniques parallel each other in their ability to index larval movements. Bright light-emitting diode (LED) lights used in optogenetic experiments tend to saturate the detectors of the camera unless filters are used and different filters maybe necessary depending on the LED spectrum and sensitivity of the camera. Impedance measures are independent of the type of LED or brightness. We also assessed the use of a non-solvent based glue (3M Vetbond) to hold larvae in place while measuring synaptic function of neuromuscular junctions, cardiac function and influence of modulators, or activation of light-sensitive channels.

## 1. Introduction

*Drosophila* larvae serve as a research model for many biological processes (i.e., immune responses, synaptic function, and development) at various levels (i.e., gene regulation and cellular to whole tissue function) [1,2,3]. Activity induced gene and protein expression in altering synaptic formation and neural connections continues to be actively pursued using larval *Drosophila* [4,5]. There are several pioneering studies in *Drosophila* which led to understanding biological principles in other animals such as humans (i.e., genetics, immune response, segmental development, and transient receptor potentials) [6,7,8,9]. Some of the findings have directly aided human health through the knowledge of how defective genes and proteins relate to disease states [10,11,12] and cardiac abnormalities [13,14].

Techniques to measure changes in behavior and physiological process allows one to have an index for addressing the many varied experimental procedures and manipulations utilized in *Drosophila* biology. Measures sometimes require costly equipment, but many measures can be made with inexpensive equipment and still aid in gathering valuable data for scientific discoveries. Body movements and cardiac function are two common measures in the field to assess mutations and perturbations related to diet, with environmental rearing and pharmacological assays on function. Recording larval movements generally are made with recording movies and then digitally tracking the moments for quantification with commercial software [15]. However, with the use of the TriKinetics *Drosophila* Activity Monitor, one is now able to also monitor activity of larva when they crawl past a light beam [16]. This technique can measure activity of locomotion and position within a tube but does not detect if a larva stops or moves its body until the larvae crosses a light beam placed in the tube. TriKinetics produces devices with multiple detectors so a fine resolution in distances and locations traveled can be detected within a narrow tube. Electrical measures of a media, such as an impedance measure, can provide a fine resolution of movement such as body wiggling or head wagging without crawling.

In some cases, the measures in dark are preferable such as with the use of light-sensitive ion channels common now in the optogenetic field of research or measure movements during a dark cycle in circadian experiments. Also, when using light-sensitive chemical agents (i.e., acetylcholinesterase (AChE) inhibitors; nitroprusside sodium) one may desire to examine behavioral and physiological effects in the dark while feeding larvae or examining the action on cardiac function [17,18].

The surge in optogenetic research has revolutionized the way one can ask questions about how identifiable neurons within neural circuits can regulate or have a role in controlling behavior the whole animal [19]. Optogenetics uses gene expression of light-sensitive proteins that serve as ionic channels or pumps which can then be activated with light [20]. Studies using light-sensitive channels are gaining ground in potential treatment for cardiac dysfunction to neurological problems such as epilepsy and Parkinson’s [21,22,23,24,25]. The ease in amenable genetic expression of these proteins in identified cells (i.e., cardiac, sensory neurons, and dopaminergic-containing neurons) is an asset of the *Drosophila* model. Being able to tightly control the lighting and background illumination in light-sensitive experimental paradigms can be an issue. Many problems are overcome somewhat by using IR light sources and IR-sensitive cameras to monitor behavioral movements. However, filters sometimes must be used for the camera because when the light-emitting diode (LED) light is on it will saturate the sensor and limit the ability of the camera to record the larval responses. Alternative approaches to record a movement or lack of could be beneficial in some cases such as using the impedance technique presented in this report. 

The principle of impedance measures is to detect a change in the voltage between two leads as the resistance changes while passing a current. Impedance measures are used in various ways such as respiratory breathing rates with expansion and relaxation of a chest for mammals [26] and clinical neuromuscular disease research [27]. Depending on how the measures are made they can be noninvasive, such as just a strap around the chest of an animal. Alternatively, two leads in a solution with an organism or a tissue placed within the small electrical field can be used. Herein, we assessed the use of impedance technique to measure larval body movement within a small chamber and to detect heart rate in dissected larvae bathed in physiological saline. Both measures can occur in the absence of light but do require one to use light to set up the recording conditions. 

Live imaging of ionic concentrations such as Ca^2+^ or Na^+^ fluxing across motor nerve terminals or within subsets of neurons in the larval brain of *Drosophila* can prove technically challenging depending on the apparatus to be used to hold the larvae stationary. This is particularly the case for dissected preparations in which insect dissecting pins may be used to hold the preparation open and in a flattened position. The use of a high-power objective lens allows a very narrow distance between the specimen and the lens. Dissection pins can be a nuisance and potentially scratch the costly lens as well as limit moving the objective lens over the full extent of the dissected larvae. Electrophysiological measures when an electrode is used to stimulate a segmental nerve, while at the same time recording membrane potentials in muscle become cumbersome with mounting pins. Researchers have overcome some of these obstacles with careful placement of the pins to hold preparations in a particular manner knowing how the electrodes will be arranged for stimulating and recording. However, when a focal macropatch electrode needs to be placed over a visualized varicosity to record synaptic responses the visual field is very narrow. This is due to the need of a water immersion objective to enhance the use of Nomarski optics to visually see the placement of the lumen of the electrode over identified nerve terminals [28,29]. The freedom to place the macropatch electrode in various locations and making use of a water immersion objective without concern of hitting mounting pins provides some ease on conducting such experiments. 

Gluing techniques are commonly used to hold adult flies or whole larvae in place; however, dissected preparations offer an additional challenge to dissect and hold the preparation in place. One concern is the effect of the glue and solvent on the physiology of the preparation. Here, we assessed the use of a non-solvent-based glue to hold larval *Drosophila* in place while measuring synaptic function of neuromuscular junctions as well as cardiac function and influence of a modulator (i.e., serotonin) on heart rate. Gluing appears to be a preferred approach for physiological measures in small organisms such as *Caenorhabditis elegans* and it works well [30].

Presenting these techniques to the scientific community has significance as it serves to help researchers with a wider range of potential tools/techniques available to them for their potential needs and serves as an obtainable and citable resource for researchers. Duplicating set-ups could be helpful for high output screening of pharmacological agents or mutant screens.

## 2. Methods

### 2.1. Larvae Expressing Light-Sensitive Channels

The filial 1 (F1) generations were obtained by crossing virgin females of UAS-ChR2-XXL (BDSC stock # 58374) with male, non-stubble 24B-Gal4 (III) (BDSC stock # 1767). When the ChR2-XXL is expressed body wall muscles are targeted. Activating CHR2-XXL results in body wall muscle contraction, leaving the larvae in a state of paralysis. The heart rate will speed up and may even stop depending on the level of channelrhodopsin activation and duration [31,32]. The rational to use the ChR2-XXL expression is that this particular form of channelrhodopsin is very sensitive to blue light and was usefully to illustrate the proof of concepts presented for these techniques 

All trans-retinal (ATR; Sigma-Aldrich, St. Louis, MO, USA) was diluted in standard fly food to a final concentration of 1 mM (for ChR2 use) and protected from light with aluminum foil. The ATR food mixture was left with a cotton plug at 4 ^°^C to allow evaporation of the alcohol solvent from the mixture. The all-trans-retinal is used as a cofactor for the channel rhodopsin which increases its sensitivity to light and increases single channel conductance [33]. After the genetic cross, early 2nd instar larvae were removed from standard food vials and placed in ATR-food mixtures and left for 48 h prior to testing. It has been noted that larval development slows in the presence of ethanol, so precautions were taken to limit its developmental influence by evaporating the alcohol [34].

Movies to illustrate the larvae movements before and during LED exposure were made with IR camera, in which the camera provided the IR light source. The particular camcorder used (Panasonic 4K video Camera Model HA-VX870 recorded at 72Mb per second for 3840 × 2160 resolution) was able to rapidly adjust to the light sensitivity from the LED to the IR lighting which recorded activity when the blue LED light was off. 

Activation of the channelrhodopsin for larval movements by blue light (470 nm wavelength, LEDsupply, LXML-PB01-0040, 70 lm @ 700 mA) was provided with a high intensity LED. The photon flux (number of photons per second per unit area) was measured with a LI-COR (model Li-1000 data Logger, LDL 3774; LI-COR from Lincoln, Nebraska, USA). This produced ~103 µMol s^−1^ m^−2^ per µA (or 22.24 µW mm^−2^) on the surface of the dish for the behaviors. As for the channelrhodopsin activated in the cardiac study, the high intensity LEDs were blue light (470 nm wavelength, LEDsupply, LXML-PB01-0040, 500 mA) and produced 101.3 W/m^2^ on the heart in these studies. The photon flux (number of photons per second per unit area) was obtained with a LI-COR (model Li-1000 data Logger, LDL3774; LI-COR from Lincoln, NE, USA), which measured µMol s^−1^ m^−2^ µA^−1^. In addition, the full spectrum of the lights was measured with a Jazz (Ocean Optics Inc., Largo, FL, USA) to obtain a total W/m^2^ from 340 to 800 nm spectrum for this light source. 

### 2.2. Impedance Measures of Larval Movement

**Figure 1:**
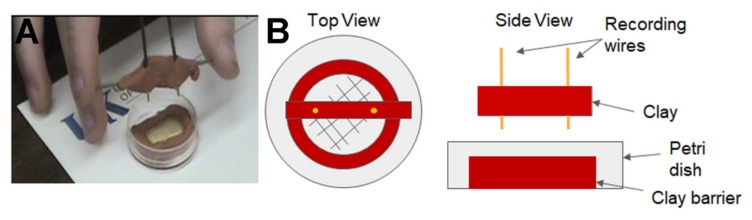
Recording dish for larval movements. Two leads for impedance measures were placed in the thin coating of apple juice. The dish is etched to provide a rough surface for larvae to grip. The clay boundary tends to restrict the larval movement to the apple juice media. (**A**) A photo of the dish used. (**B**) A top and side view in schematic form with the parts labeled.

For electrical recordings, two wires were connected to an impedance detector (UFI, model 2991), which measure dynamic resistance between the leads monitoring the movements on the recording dish. The detector was linked to a PowerLab/4SP interface (AD Instruments) and calibrated with the PowerLab Chart software version 5.5.6 (AD Instruments, Australia). All recordings were made with a 10 kHz sampling frequency. The other ends of the two wires had soldered gold coated pins and the tips were placed in the shallow layer of apple juice in which the larvae were placed (Figure 1). This is explained in video format (**Movie 1:** procedures of monitoring larval movements with impedance; https://youtu.be/b5UbKfyGrh4).

When the larvae move a displacement of the current field between the two wires in the apple juice occurs. This presents a change in the resistance in the field and measured as a displacement in voltage. The digital responses are recorded with the software which can be examined for larval movements.

### 2.3. Impedance Measures of Larval Heart Beats

**Figure 2:**
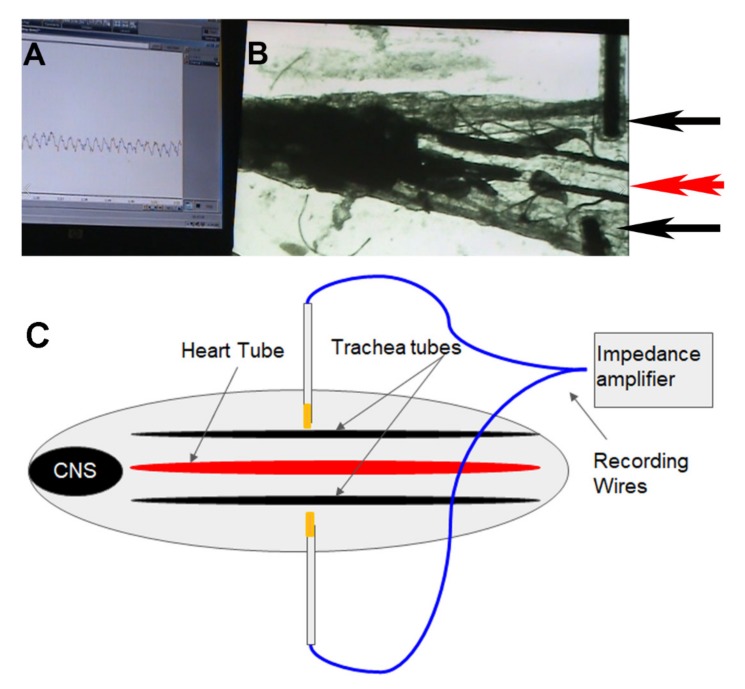
Simultaneous recording of impedance and video of heartbeats. (**A**) Electrical measure of impedance. (**B**) Video recording shown on computer screen. Single arrow heads indicate the leads place on either side of the heart tube in a dissected preparation. The double arrowhead points to the heart tube between the two tracheal tubes. The beats on the computer screen match the direct observation of the heart beats. (**C**) A schematic of the larva with the impedance recording set up.

Only early 3rd instar larvae were used (50–70 h post hatching). This stage was easily identified due to the size of the *Drosophila* larvae (~5 mm). The dissection to expose the heart tube is relatively feasible without damage. Larvae were maintained at room temperature ~21 ⁰C in vials partially filled with a cornmeal–agar–dextrose–yeast medium. For using larvae expressing channelrhodopsin, early 2nd instar larvae were removed from standard food vials and placed in ATR-food mixtures and left for 48 h prior to testing. The dissection of the larvae to expose the heart tube is detailed in text and video format as an open source format [35]. In brief, the larvae were dissected ventrally and pinned on four corners. The visceral organs were removed keeping the heart tube intact. This dissection technique was previously used to directly assess pharmacological agents on the heart of *Drosophila* larvae [36,37,38]. The dissection time was ~3–6 min, and the muscles were allowed to relax while bathed in saline for 3–5 min after dissection. To examine the ability to use the gluing technique of liquid of Vetbond, the dissection above was used and then the saline was removed and a small amount of Vetbond from a toothpick was placed by the head region where it was originally pinned. With the dissection pin, the head of the preparation was slid over the Vetbond drop. The cuticle and Vetbond adhered the larval head to the spot on the glass slide. This was repeated for the caudal end, where the two pins were holding the body cavity open. The pins were removed, and the dissection dish is placed in view for the camera and set up for impedance measures. The novelty is illustrating the ability to monitor with electrical recordings the beating heart tube while glued. Two wires were connected to an impedance detector as mentioned above for monitoring larval movements. Here the two leads were insulated stainless steel wires (0.13 mm diameter; A–M Systems, Carlsburg, WA, USA) and placed inside the body wall boundary on both sides of the heart tube (Figure 2). The insulation was kept to the end of the wires, only exposing the cut ends of the wires. Care was taken not to pull on the tracheal tubes when placing the wires as accessory muscles attach the heart tube to the trachea. In this illustration the lighting in the room was turned off and dim white light was illuminating the preparation through the microscope base to allow the preparation to dark adapt for later activation of the channelrhodopsin to access the effect on the heart rate while monitoring with the impedance technique.

### 2.4. Recording Synaptic Responses in a Larva Glued to a Glass Slide

**Figure 3:**
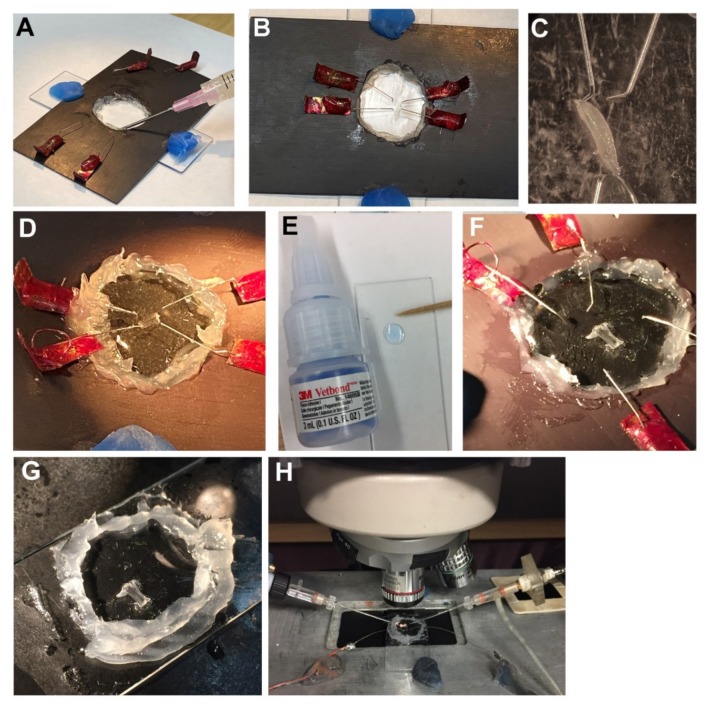
Gluing a dissected larva to a glass slide for imaging or for electrophysiological recordings. (**A**) The magnetic strip was sealed to the glass slide with petroleum jelly and wax. The petroleum jelly keeps saline from spreading under the magnet strip. (**B**) The dissecting pins were put in place to then pin the larvae. (**C**) The larval head and caudal end were pinned with the dorsal side facing up. The larva was cut along the length of the larvae and the internal organs were washed away by exchanging the saline and cutting excess tissue. (**D**) The larva was now ready to be glued to the dish after it is spread out in the four corners. (**E**) A drop of Vetbond was applied to a glass slide and a toothpick was used be used to take a small amount and place just next to the cuticle of the larva to be glued. A very thin layer was used to adhere the cuticle. (**F**) The four dissection pins are removed after the corners are glued. (**G**) Saline is added, and the petroleum jelly ring is repaired to hold the saline. (**H**) The dissected larva was then placed on the microscope stage for physiological recordings.

The larval dissections were performed as described in detail previously [39,40] for pinning ganglia isolated from the leech ventral nerve cord [41] with the exception of mounting the larvae to a removable glass slide. The dissection dish used herein was mounted onto a glass slide, using petroleum jelly to seal the glass slide to the magnetic strip so saline would not leak under the magnetic strip (Figure 3A). Wax was also used to hold the slide to the magnetic strip. A hole in the center of the magnetic strip allowed the preparation to be viewed with transmitted light. Dissecting pins were bent and glued to paper clips and coated with fingernail polish as to prevent rust so they could be used repetitively. The dissection pins need to be prepared earlier and allowed to air dry prior to use. Paper clips are maneuvered easily on the magnetic strip to hold the fillet preparation in place. 

After the dish was sealed with the glass slide and magnetic strip, the pins were moved into position (Figure 3B) and a larva was placed on the dish (Figure 3C). The larva was checked to ensure the dorsal aspect is facing up and then the head and tail were pinned down. The pins are moved by sliding them along the magnet strip. Saline was then added, and the preparation was slit along the mid-dorsal longitudinal axis with fine scissors. The use of saline helps to float the intestines and salivary tissues so it can be washed away by exchanging the saline a few times. The segmental nerves were cut close to the brain and the brain was removed along with the internal organs. The preparation was then stretched out leaving the cuticle and body wall muscles along with the segmental nerves freely floating in the saline (Figure 3D). The liquid of Vetbond was placed on a separate glass slide and a toothpick was used to obtain a small amount liquid (Figure 3E). With a tissue paper to serve as a wick, the saline was removed and a dissecting pin in a corner was lifted while pushing that corner toward the midline. The small amount of Vetbond from the toothpick was placed where the corner was pinned originally. With the dissection pin, the corner of the preparation was slid over the Vetbond drop. This would adhere the cuticle of the larvae to that spot. This was repeated for the other three corners of the dissected preparation in a quick manner (Figure 3F). Next, the pins were removed and the magnetic strip lifted off the glass slide. The petroleum jelly wall was repaired with additional petroleum jelly. Then saline was reapplied over the preparation surrounded by the boundary of petroleum jelly (Figure 3G). The saline was exchanged one time. The larval preparation was then placed on a recording stage for monitoring synaptic responses (Figure 3H).

**Figure 4:**
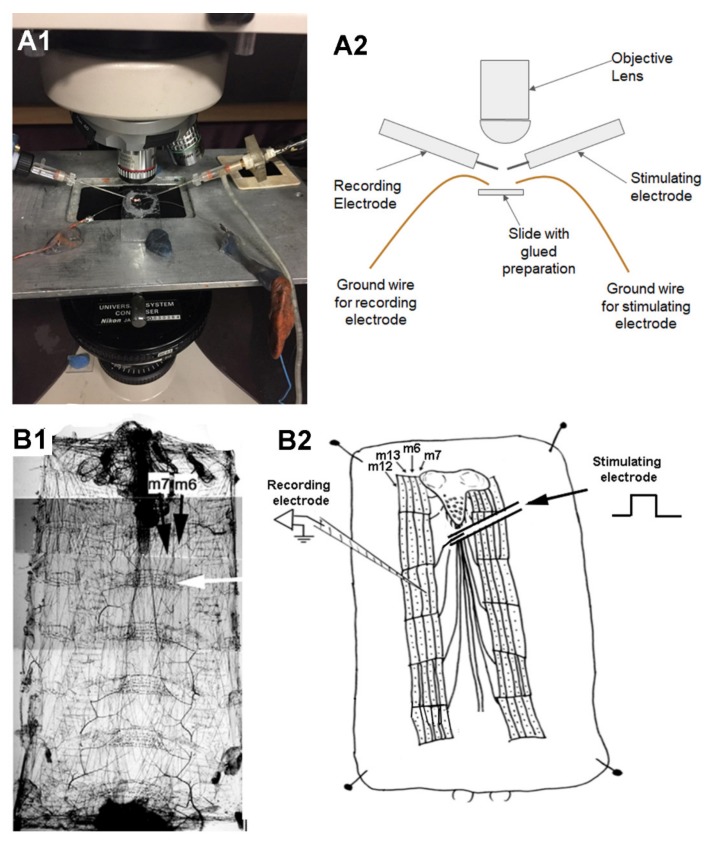
Physiological recordings of synaptic potentials in a glued larval preparation. The petroleum jelly border is adjusted as needed to provide access for the stimulating suction electrode for the nerve and the intracellular microelectrode. Ground wires for the stimulating and recording arrangements are placed into the saline bath over the petroleum jelly ring. (**A1**) A photo of the recording arrangements and (**A2**) a schematic listing the components of the set up. (**B1**) Is the dissected larva. The white arrow depicts a segmental boundary as seen with the horizontal banding. (**B2**) A schematic illustrating a suction electrode stimulating the segmental nerves to the muscles. Muscle m6 was recorded with an intracellular electrode.

To record synaptic responses in a muscle fiber, the 3rd segmental nerve of interest was pulled into a suction electrode and stimulated (Figure 4). The larval body wall muscle m6 was used to monitor the transmembrane potentials with sharp intracellular electrode (30 to 40 megaOhm resistance) filled with 3 M KCl. An Axonclamp 2B (Molecular Devices, Sunnyvale, CA, USA) amplifier and 1 X LU head stage was used. The excitatory junction potentials (EJPs) were collected with LabChart 7.0 (ADInstruments, USA) as previously detailed [42]. Fly saline hemolymph-like 3 (HL3) [40,43] was used: (in mmol/L) 70 NaCl, 5 KCl, 20 MgCl_2_, 10 NaHCO_3_, 1 CaCl_2_, 5 trehalose, 115 sucrose, 25 N,N-bis(2-hydroxyethyl)-2-aminoethane sulfonic acid (BES), and pH at 7.1. As a proof of concept in the viability of the preparation, physiological recordings were made before and during the exchange of saline containing bacterial lipopolysaccharides (LPS) *Serratia marcescens* at 500 µg/mL. The LPS was left for one minute prior to exchanging the bath back to normal saline. 

## 3. Results

### 3.1. Larval Movements with Impedance Detection

**Figure 5:**
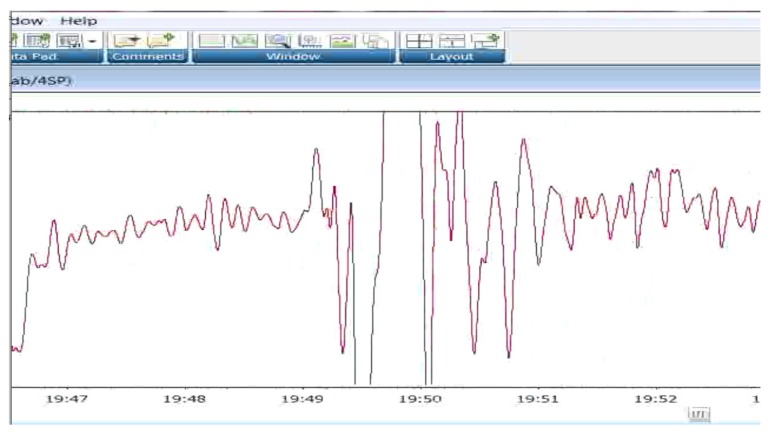
Impedance measures of when the larva are moving in the recording dish. This is a screen shot of the computer screen using the Chart software to record the deflections in the impedance signal.

The larval movements in the apple juice containing plate are readily detected with the impedance measure and correlate with visual measures. When the channel rhodopsin channels were activated with blue LED light, the larva decreased and stopped moving. The impedance signals matched the visual observations recorded with the camera. Large movements can produce large defections and smaller movements such as a head wag produce smaller ones as seen in Figure 5. After the LED light was turned off the larva starts to recover with movements and the deflections in the impedance measures returned. A movie is provided to illustrate the impedance detection while a larva was moving. One movie is the recording made with the video camera (**Movie 2**: https://youtu.be/sBTGScDt-P8) and another movie (**Movie 3**: https://youtu.be/XWAHaQ3zgg4) is depicting the small screen on the video camera as well as the computer screen monitoring the impedance signals.

### 3.2. Impedance Measures of Heart Rate

The visual measures of the heartbeat directly match the measures obtained with the impedance technique and are able to be recorded without light (**Movie 4**: Recording heart rate with impedance measures; https://youtu.be/kK6g0FboASs). Speeding up the heartbeat with activating the channelrhodopsin expression with blue light was also directly measured with the impedance and visual recordings. The rates match the two measures in synchrony. A movie is provided to illustrate the impedance detection while altering the heart rate by activating the light-sensitive channels expressed in the heart tissue. This illustrates the proof of concept in using impedance to detect heart rate and changes in the rate by activating the light-sensitive channels expressed in the heart. In addition, this also illustrates the proof of concept of using a larval preparation that was glued in place. 

### 3.3. Synaptic Measures at NMJs of a Glued Larva

**Figure 6:**
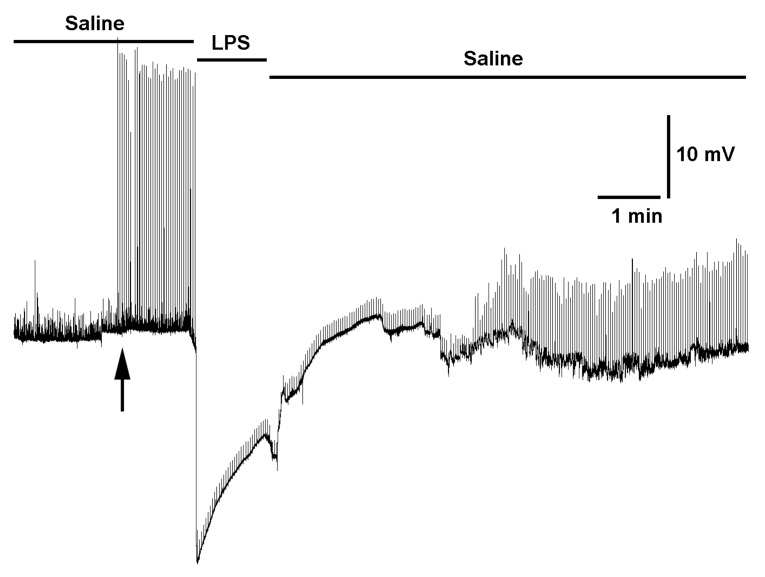
Synaptic response of excitatory junction potentials (EJPs) in a larva using the glued technique. Response recorded in m6 of segment 3. Initially, the recording shows spontaneous quantal events. Then the motor nerve was stimulated at 0.5 Hz as indicted by the arrow. The saline bath was exchanged for bacterial lipopolysaccharides (LPS) *Serratia marcescens* at 500 µg/mL and left for 1 min prior to exchanging the bath back to normal saline. The evoked EJPs start to recover during the saline wash out.

Synaptic recordings of the excitatory junction potentials (EJPs) made in muscle 6 in segment 3 of a dissected larva which was glued to the dish. The recordings were just as robust as for preparations that were only pinned and not exposed to the gluing technique (Figure 6) [42,44,45]. Recordings made before and during the exchange of saline containing bacterial lipopolysaccharides (LPS) *Serratia marcescens* at 500 µg/mL and left for 1 min prior to exchanging the bath back to normal saline. The evoked EJPs start to recover during the saline wash out. This result replicates prior studies reporting on these relatively recent novel findings in the action of LPS on synaptic transmission at larval Drosophila NMJs [42,44,45]. LPS is noted to cause a rapid (<1 s) transit hyperpolarization of the body wall muscles and a depression in the amplitude of evoked EJPs and spontaneous quantal events. The depression in the glutamatergic synaptic transmission is due to the blockage of glutamate sensitivity on the muscle fibers.

## 4. Discussion

Our hope is that presenting these techniques, which we have found useful for conducting various experiments with larval *Drosophila*, will also benefit other researchers in the field. This technique could also be expanded on for other small organisms to record bodily movements or potential heart rates depending on the arrangement of the organ and recording leads. We have recently been able to monitor movements of aquatic planaria with this technique (**Movie 5**: Planarian movement recorded with impedance and IR camera; https://youtu.be/eSGO4Nis1lE). With the increasing application of optogenetic approaches used in larval *Drosophila* related to monitoring movements the impedance technique adds another approach possible. Determining a recovery time from the larvae being paralyzed can be obtained for various experimental paradigms using this approach in the absence of white light background for a recording by a camera or direct visual inspection. We found the electrical measures of impedance an easier approach than having a camera continuously record to wait for a movement to occur. The electrical activity can be screened on a computer file to when deflections in the baseline occur. The use of the impedance measures has limits, as one does not know what type of movement is being made. Thus, it depends on the experimental questions one is asking related to behavioral measures as for which techniques are most suitable.

As for recording heart rate, the electrical measure allows the analysis to be easily automated by measure in the rhythmic deflections instead of more difficult to use automated image analysis or the time-consuming task of direct visual counts from recorded video. In addition, in the case of the intense blue LED lights for optogenetic studies one should not be looking at the lights as retinal damage can occur. Although specialized filters could be implemented for direct visualizing of counting the beats, there is no permanent record as with captured electrical impedance signals. No reports have been forthcoming on the effects of small electric fields on larval *Drosophila* imposed by impedance measures. Long-term studies in larval movement or possible even for monitoring tissue movements such as the heart, could be affected in the electrical filed is strong. This remains to be examined for future studies. The commercially bought impedance amplifiers used in these studies does have the ability to increase the gain which is related to the amount of current applied between the two leads. The current provided in this case is from a standard 9 V battery (Duracell 9 Volt alkaline battery-MN1604 Coppertop), which has the capacity of 580 mAh. We would suggest using the smallest gain setting to detect movements of interest. The current field will depend on the media used in which the measures are to be made.

The use of the non-solvent-based Vetbond has helped immensely in being able to use water immersion objective lens over dissected preparations for ionic flux measures in live imaging of nerve terminals. The delicate balance of arranging dissection pins around the movements of the objective lens is removed by use of the gluing technique. It is surprising this technique has not been more widely use with larval *Drosophila* as it is commonly used for *Caenorhabditis elegans* studies [28]. In addition, not having to plan for which way the dissecting pins are arranged for electrophysiological measures has provided more freedom in the placement of the electrodes and the recording dish on the microscope platform.

Some of the advantages and disadvantages we have incurred with these techniques are highlighted as follows.

Disadvantages of impedance technique for measuring movements:

1. Need to place wires in moist media for detecting body wall movements.
2. Animals crawl out of dish. Maybe use “ant farm” technique with glass slides and moist food.
3. Small distance between wires easier to detect signals. How small of a movement can be detected has not been determined.

Advantages of impedance technique for measuring movements:

1. No light is required during the measures. Good for optogenetic studies for light activated channels.
2. Can be used for body movements as well as heart rate monitoring.
3. Can be used in cold or incubator at different temperatures (i.e., no fogging of camera lens).

Disadvantages of gluing technique:

1. Need to use small amounts of glue.
2. Can glue mouth closed or position larvae in wrong position and cannot reposition.
3. Sometimes glue stick is attached to larvae if touch larva by mistake.
4. Glue dries out prior to getting larvae stuck and taking too long to glue preparation down while reducing the saline around preparation.

Advantages of gluing technique:

1. Insect pin removal allows freedom to place electrophysiological electrodes in various positions without pins interfering.
2. Much easier for focal macro-patching over nerve terminal varicosities.
3. Close positioning of objective lens for imaging of motor nerve terminals in live preparations.
